# Supplementary material for: Dynamic transcriptomic profiles of zebrafish gills in response to zinc supplementation
Source: BMC Genomics. 2010 Oct 11;11:553. doi: 10.1186/1471-2164-11-553 (PMC3091702; doi:10.1186/1471-2164-11-553)
Supplement: Additional file 2 — Interactive Direct Interaction Network representing the molecular interactions between zinc, copper, iron, calcium and proteins encoded by transcripts changed by zinc supplementation. Mini web-site containing index.html and hyperlinked pages in subdirectory describing a Direct Interaction Network automatically generated based on curated interactions contained within the proprietary PathwayArchitect database. Ovals represent proteins and the circles symbolize metal ions. Objects are coloured by their abundance in zebrafish at the time-point they were significantly different from the control is a scale from -4 fold (dark green) to +4 fold (dark red). Where significant differences were found at more than one time-point, the colour overlay shows expression at the first instance. Dark blue squares denote 'binding', and light blue squares 'expression'; green squares stand for 'regulation', green diamonds for 'metabolism', and green circles for 'promoter binding'. Arrow heads indicate directionality of the interaction where annotated. All nodes and edges can be further interrogated by selecting the relative area of the image. [file 1471-2164-11-553-S2.zip › PathwayArchitect Zn xs DIN/437687.html]

# REGULATION:

|  |  |
| --- | --- |
| Type | REGULATION |
| Effect | None |


---

|  |  |
| --- | --- |
| Score | 0 |


---

|  |  |
| --- | --- |
| Reference Count | 3 |


---

|  |  |
| --- | --- |
| Mechanism | Unknown |


---

|  |  |
| --- | --- |
| Reference:0 || Sentence | "The SERCA2 isoform of the Ca2+-ATPase represents a major Ca2+-removal system in cardiac muscle fibres and phospholamban is a regulator of Ca2+-pump activity." |
| PMID | 11029519 |
| Year | 2000 |
| Species | Human |
| Journal | Int J Mol Med |
| RefScore | 1 |
| Source | PArchNLP |
  |
|  |  |


---

|  |  |
| --- | --- |
 Reference:1 || Sentence | "We used the tachycardia-induced canine model of HF and human failing hearts to investigate the density and major functional properties of RyRs, SERCA2a, and phospholamban (PLB), the main proteins regulating SR Ca2+ transport." |
| PMID | 12456487 |
| Year | 2002 |
| Species | Human |
| Journal | Circ Res |
| RefScore | 0 |
| Source | PArchNLP |
  ||  |  |


---

|  |  |
| --- | --- |
 Reference:2 || Sentence | "The inotropic/lusitropic effects of beta-adrenergic agonists on the heart are mediated largely by protein kinase A (PKA)-catalyzed phosphorylation of phospholamban, the natural protein regulator of the Ca2+ pump present in sarcoplasmic reticulum (SR) membranes." |
| PMID | 10381780 |
| Year | 1999 |
| Journal | J Pharmacol Exp Ther |
| RefScore | 1 |
| Source | PArchNLP |
  |


---

|  |  |
| --- | --- |
